# Durable response to pembrolizumab in hepatic metastasis from colonic carcinoma with Lynch syndrome: a case report

**DOI:** 10.3389/fimmu.2024.1455907

**Published:** 2024-08-23

**Authors:** Cheng Xue, Dongqing Zhu, Xin Wang, Lina Jiao, Yunhui Lu, Sanli Zhang, Jiayi Lv, Linlin Cui, Mengna Ruan, Dechao Xu, Qingyang Liu, Yun Feng, Shuqin Mei

**Affiliations:** ^1^ Kidney Institute, Department of Nephrology, Shanghai Changzheng Hospital, Naval Medical University (Second Military Medical University), Shanghai, China; ^2^ Department of Radiology, Shanghai Changzheng Hospital, Naval Medical University, Shanghai, China; ^3^ Department of Health Management Center, Shanghai Changzheng Hospital, Naval Medical University, Shanghai, China; ^4^ Department of Hepatic Surgery, Fudan University Shanghai Cancer Center, Shanghai, China

**Keywords:** immune-checkpoint inhibitors, Lynch syndrome, metastatic colorectal cancer, mismatch repair deficiencies, treatment related adverse event

## Abstract

Pembrolizumab and other immunotherapies have become central in treating metastatic colon cancer, particularly effective in patients with mismatch repair deficiencies. We report a case involving a man who initially underwent radical surgery for sigmoid colon cancer on April 27, 2011, followed by hepatic tumor resection on September 21, 2017. Post-surgery, he received eight cycles of adjuvant chemotherapy with the CAPEOX regimen and was regularly monitored through CT and MRI scans. On August 24, 2022, liver metastases were detected, and he was diagnosed with Lynch syndrome (LS) due to germline mutation in the *MSH2* and *EPCAM* genes. He commenced treatment with 200mg of pembrolizumab intravenously every three weeks on September 2, 2022, and demonstrated a sustained response. However, after 17 cycles, he developed a treatment related adverse event (TRAE) of pancreatic endocrine dysfunction, leading to type 1 diabetes, managed with subcutaneous insulin injections. After 30 cycles of treatment, no evidence of disease was observed. This case underscores the significant clinical benefits of first-line pembrolizumab in managing hepatic metastasis in colonic carcinoma associated with LS, despite the occurrence of TRAEs. It raises critical questions regarding the optimal duration of immunotherapy following a complete or partial response and whether treatment should be discontinued upon the emergency of TRAEs. Continued research and forthcoming clinical trials with checkpoint inhibitors are expected to refine treatment protocols for LS-associated carcinoma.

## Introduction

Pembrolizumab is the classic immune checkpoint inhibitor (ICI) and has various roles in advanced malignancies including the colon, lung, kidney, and bladder as well as lymphomas and melanoma. The unexpected response rate accelerates the approval of pembrolizumab monotherapy by US Food and Drug Administration (FDA) in 2017 (1).

Mismatch repair deficiencies (dMMRs) are predictive of response to immunotherapy in metastatic colorectal cancers. Because of the dMMRs, there are more mutations in DNA to pass unrepaired during cell division, thereby promoting tumor growth. The most familiar mismatch repair genes include *MLH1*, *MSH2*, *MSH6*, *PMS2*, or *EPCAM* (deletions of the epithelial cell adhesion molecule gene). It is reported that *MSH2* or *MLH1* accounts for almost 60% to 80% of all Lynch syndrome (LS)-associated cancers, others are attributed to *MSH6* or *PMS2*, rarely, to *EPCAM (* 2). A small subset of patients accumulate transcriptional errors in regions with repetitive nucleotide sequences throughout the genome (microsatellite instabilities, MSI). This microsatellite unstable phenotype is termed dMMRs/microsatellite instability-high (MSI-H).

LS is one of the most prevalent hereditary cancer syndromes and accounts for some 3% of unselected patients with colorectal or endometrial cancer and 10–15% of those with DNA mismatch repair (MMR)-deficient tumors. Extracolonic malignancies are also common, including endometrial, which is the most common, but also tumors of the upper gastrointestinal tract, urinary tract, ovarian, hepatobiliary tract, pancreas, and brain (3). It is characterized as an autosomal dominant cancer genetic predisposition syndrome due to a germline mutation in one of the mismatch repairs mentioned above.

Although dMMRs/MSI-H are new indications for pembrolizumab, our experience with it and other checkpoint inhibitors in MMR-deficient tumors is still limited. Herein we report a case of a patient with hepatic metastasis of colonic carcinoma and LS due to *MSH2* and *EPCAM* genes mutation who is enjoying an ongoing, prolonged treatment response of almost two years to single-agent pembrolizumab, even though he underwent treatment related adverse event (TRAE) during the treatment period of monotherapy.

## Case description

A 60-year-old Asian man with a 15-year of hypertension and no other past medical history, initially presented in April, 2011. He had bright red blood per rectum (BRBPR) for months initially attributed to hemorrhoids and about 5kg weight loss during the past half a year. His family history was positive for colon cancer in his mother (diagnosed in the 50s) and one sister died of colon cancer in her 20s. He underwent a colonoscopy examination and was diagnosed with sigmoid carcinoma. He was surgically staged as Stage IIA (T3N0M0, with no metastatic disease on subsequent computed tomography (CT) imaging). The postoperative pathological assessment revealed mucinous adenocarcinoma localized to the sigmoid colon. He received a course of adjuvant chemotherapy with eight cycles of adjuvant chemotherapy following the CAPEOX regimen, involving Oxaliplatin (130 mg/m^2^ IV on day 1) and Capecitabine (1000 mg/m^2^ PO twice daily for 14 days every three weeks), which was complete in October 2011. Despite a family history suggestive of colorectal cancer predisposition, genetic testing for dMMR and MSI-H was not performed at the time of the initial diagnosis. This was partly due to the limited availability and awareness of genetic testing for LS in China in 2011.

In the intervening years, he underwent routine surveillance follow-ups with no evidence of disease on annual clean colonoscopies every year and magnetic resonance imaging (MRI) until October 2016.

**Figure 1:**
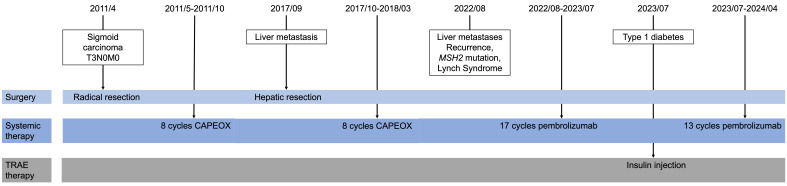
Timeline in case presentation.

In September, 2017, he was diagnosed with hepatic tumor and underwent hepatic tumor resection, disclosing postoperative pathology indicative of metastatic adenocarcinoma. Immunohistochemistry demonstrated deficient mismatch repair proteins, with loss of both *MSH2*, *MLH*, and *MSH6*. In the aftermath of the surgical intervention, an additional eight cycles of CAPEOX regimen were administered (**Figure 1**).

**Figure 2:**
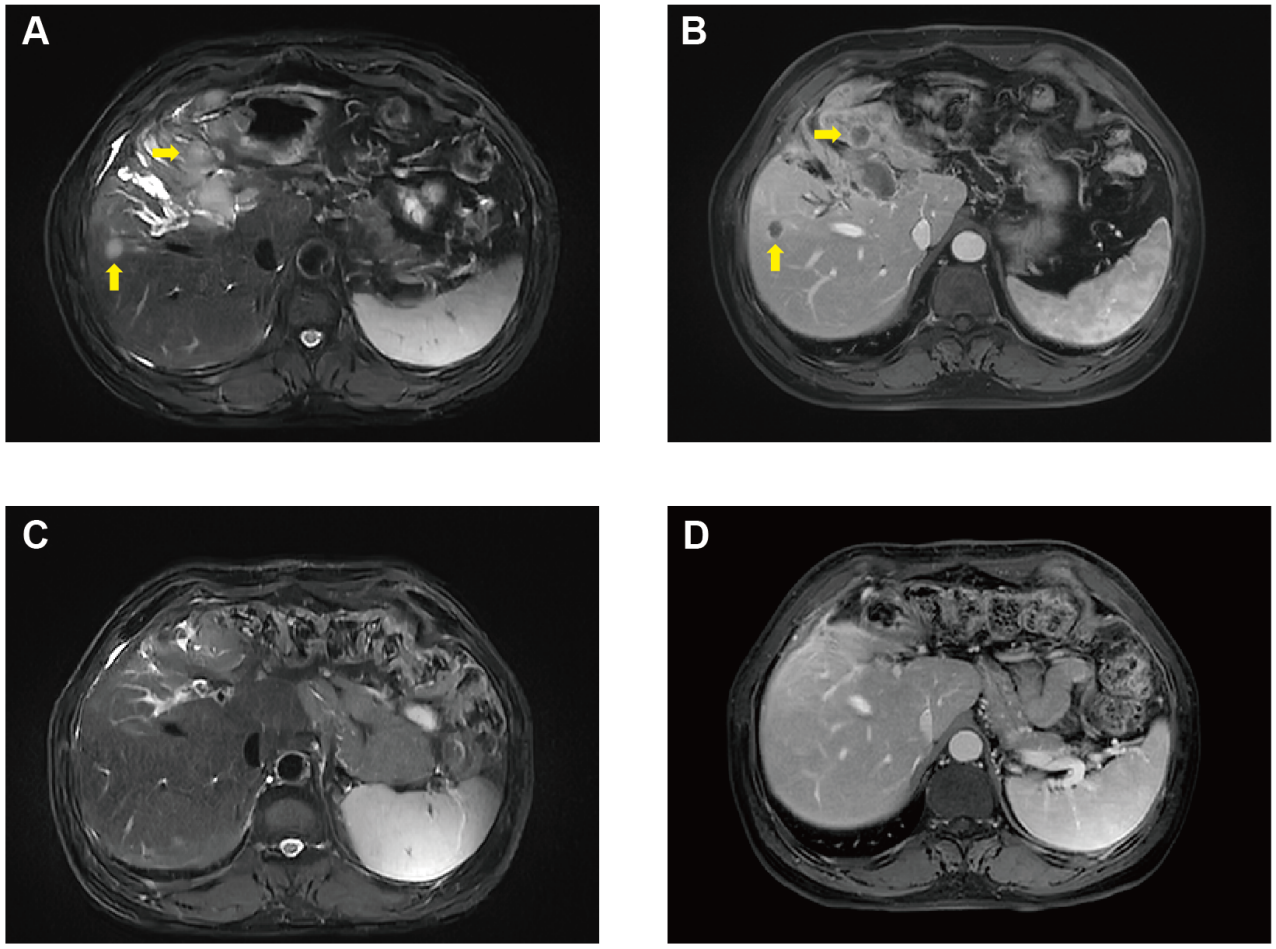
MRI exam changes before **(A, B)** and after **(C, D)** ICI therapy.

**Figure 3:**
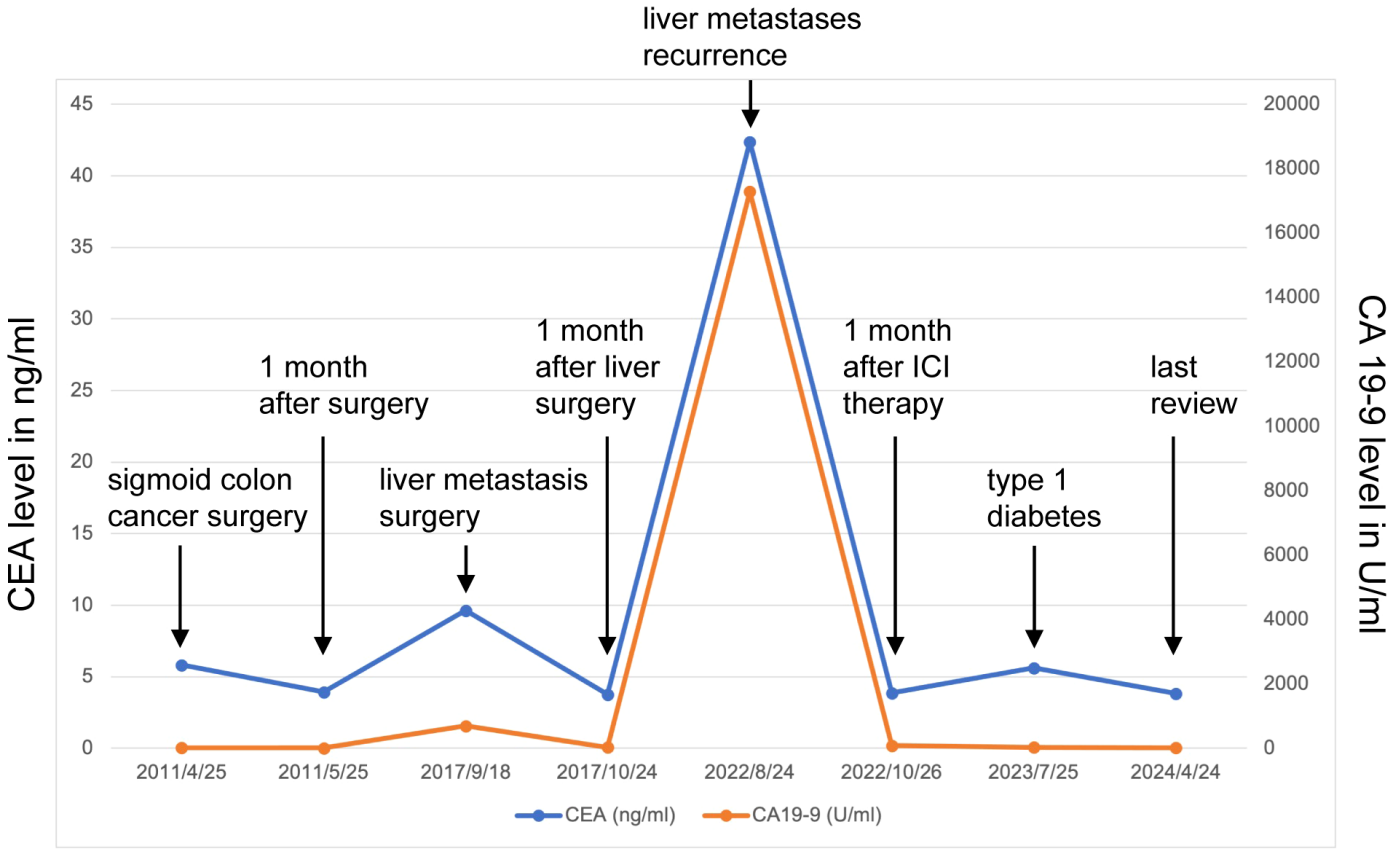
CEA/CA19-9 level changes before and after ICI therapy.

Unfortunately, in August 2022, he was diagnosed with liver metastasis with abdominal MRI on routine physical examination, which unveiled a spectrum of intrahepatic complexities, including multiple metastatic lesions, intrahepatic bile duct dilation, metastasis to the porta hepatis lymph nodes, and a potential tumor thrombus in the left branch of the portal vein (**Figures 2A, B**). Physical examination was unremarkable. Laboratory results showed an evidently increase of carbohydrate antigen 19-9 (CA19-9) and carcinoembryonic antigen (CEA) (**Figure 3**), which were 17288U/ml (0-39) and 42.37ug/L (0-5), respectively. The alpha fetal protein (AFP) was normal with 2.7ug/L (0-20). Further information revealed that one of his brothers developed carcinoma of the bladder in his mid-50s and died of glioblastoma. Another brother developed colon cancer in his 60s. His niece also developed colon cancer and papillary thyroid carcinoma in the past 5 years. Ultimately, he was diagnosed with LS based on germline testing done on peripheral blood DNA which showed mutation in *MSH2* and *EPCAM*, with a TMB of 35.8 mutations/megabase, with no alterations in KRAS or BRAF identified. And microsatellite instability testing was positive.

Because of the known tumor MMR deficiency, he subsequently underwent immunotherapy of anti-programmed death 1 (PD-1) antibodies pembrolizumab at a dose of 200 mg intravenously every three weeks. Only after 3 cycles of treatment of pembrolizumab, the laboratory results and clinical status of the patient improved tremendously (**Figure 3**). However, after 17 cycles of treatment, he attended our clinic because of diabetic ketoacidosis. His data at onset, including extremely low serum C-peptide (0.08 ng/mL), fulfilled the diagnostic criteria for fulminant type 1 diabetes. Using multiple daily insulin injection therapy, his glycemic control has been maintained.

He continued pembrolizumab treatment and aside from immunotherapy-induced type 1 diabetes, he tolerated treatment well. He received a total of 30 cycles of pembrolizumab. CA19-9 and CEA remained within normal limits (**Figure 3**) and surveillance radiographic changes in May, 2024 showed no evidence of metastatic disease, indicating a remarkable response when compared to the MRI before the initiation of immunotherapy (**Figures 2C, D**). He was also being followed with yearly flex sigmoidoscopy, upper endoscopy every year, and oncology clinic visits with laboratory test index every nine weeks. Immunotherapy was optimally tolerated and the patient reported an active life and returned to his full-time employment.

## Discussion

We present a case of a complete metabolic and serologic remission in a patient with dMMR/MSI-H metastatic colorectal cancer (mCRC) and Lynch syndrome under treatment with pembrolizumab monotherapy after radical surgery for sigmoid colon cancer on April 27, 2011, and hepatic tumor resection on September 21, 2017, respectively. Following 8 cycles of adjuvant chemotherapy with the CAPEOX regimen after each operation.

Up to 30% of CRC are inherited, and known syndromes account for only 2-5% of all cases. The two most common syndromes are LS and familial adenomatous polyposis (FAP) (4). LS is diagnosed using clinical criteria like the Amsterdam Criteria II and Revised Bethesda Guidelines, tumor testing for MSI and immunohistochemistry to detect MMR protein deficiencies, and genetic testing of peripheral blood DNA to identify germline mutations in MMR genes (*MLH1, MSH2, MSH6, PMS2, EPCAM)*. Next-generation sequencing (NGS) enhances sensitivity and specificity. A detailed family history helps identify LS patterns, and genetic counseling is recommended (5). LS is characterized by germline mutations in the MMR genes that cause genomic instability and therefore tumorigenesis. Due to the loss of expression of these proteins, mutations in microsatellite regions (repetitive DNA sequences) accumulate, resulting in MSI and a high tumor mutational burden (TMB).

Recent advancements have unveiled new diagnostic methods. Liquid biopsy techniques analyzing circulating tumor DNA (ctDNA) offer a non-invasive diagnostic and monitoring tool, detecting MSI and MMR deficiencies from blood samples. Emerging evidence highlights the role of microRNAs (miRNAs) in LS pathogenesis, where overexpression of miR-155, miR-21, and miR-137 may induce MSI or modulate MMR gene expression. These miRNAs could serve as biomarkers and therapeutic targets, enhancing precision medicine approaches for LS. Ongoing research into the miRNAs involvement in LS provides insights into novel diagnostic and therapeutic strategies, promising to refine the management of this hereditary cancer syndrome (6).

Treatment of mCRC with dMMR/MSI-H has traditionally been challenging due to the poor response to conventional chemotherapeutic therapy, just like in our case (7). However, the long-term remissions induced by ICIs in many types of cancers have opened up the possibility of a broader use of immunotherapy in less immunogenic but genetically heterogeneous tumors. For mCRC, pembrolizumab has been approved as the preferred option and nivolumab, alone or in combination with ipilimumab as an alternative option for patients with dMMR/MSI-H disease, independently of their eligibility for intensive chemotherapy. The KEYNOTE-177 study showed that in patients with dMMR/MSI-H mCRC, pembrolizumab significantly improves progression-free survival (PFS), objective response rate (ORR), and complete response (CR) rate, doubling median PFS from 8.2 months to 16.5 months, extending ORR from 33.1% to 43.8%, and increasing CR rate from 3.9% to 11.1%, comparing with the standard chemotherapy (3). Previous studies about mCRC treated with pembrolizumab were summarized in **Table 1**, and most of the cases got CR or PR (8–17). A clinical study by Le et al. also highlighted the effectiveness of ICIs in dMMR/MSI-H colorectal cancer, corroborating our findings. Additionally, cases reported by André et al. provided evidence of durable responses and manageable adverse events of Pembrolizumab in MSI-H CRC, similar to our patient’s experience (18, 19).

**Table 1:** Characteristics of studies mCRC treated with pembrolizumab.

Author	Sex/Age	Metastases	Primary tumor/ Stage	Treatment	Germline gene pathogenic variant	MSI-H	TMB	TRAE	Outcome
Our study	M/60	Liver	Colon adenocarcinoma/ stage IIA	Pembrolizumab	*MSH2*, *EPCAM*	H	High	Type 1 diabetes	Achieved cCR within 3 months and has remained progression-free until now
Xu et al. (8)	M/43	Liver	Colon adenocarcinoma/ stage IIIB	Pembrolizumab after 4 cycles of standard adjuvant chemotherapy	*MSH2*	H	N/A	N	Achieved cCR within 2 months
Keane et al. (9)	M/26	Liver	Colon adenocarcinoma	Pembrolizumab	*MSH2*	H	High	Sarcoid-like reaction	Continue to have durable cancer control as well as stable SLR 24 months after discontinuation of ICI
Keating et al. (10)	M/33	Lung and liver	Colon adenocarcinoma	Pembrolizumab	*MSH2*, *MSH6*	N/A	N/A	Hypothyroidism	Discontinue ICI after 48 cycles and alive with improved quality of life
Krekeler et al. (11)	M/63	Prostate, liver and peritoneal	Colon adenocarcinoma	Pembrolizumab for 7 cycles, then change to nivolumab in combination with ipilimumab	*MSH6*	H	N/A	N	After a total duration of 17 months of ICI, get complete remission
Yang et al. (12)	M/64	Retroperitoneal lymph node, inferior vena cava, right ureter, kidney and right renal arteriovenous	Colon adenocarcinoma	Pembrolizumab and radiation	*MLH1*, *PMS2*	H	N/A	N	Get robust partial remission
Feng et al. (13)	M/38	Lung	Urothelial carcinoma; sigmoid colon adenocarcinoma	Pembrolizumab	*MSH2*	N/A	N/A	N	Sigmoid colon lesion was evaluated as progressive disease; ureteral lesion was assessed as PR
Salman et al. (14)	M/38	Rib and dorsal vertebra	Mucinous adenocarcinoma of cecum	Pembrolizumab	*MSH2*, *EPCAM*	H	N/A	N	This immunotherapy scheme delayed the second-line treatment with FOLFIRI and Avastin for 8 months
Patil et al. (15)	M/65	Pancreatic, liver and lung	Colon adenocarcinoma	Pembrolizumab	*MLH1*	H	N/A	N	Partial response and CT showed no evidence of new metastatic disease
Nakase et al. (16)	F/41	Glioblastoma	Rectal cancer	Pembrolizumab and radiation	*MSH2*	H	N/A	N	Died due to glioblastoma progression 19 months after the initial treatment
Djerroudi et al. (17)	M/50	Perivascular epithelioid cell tumors	Colon adenocarcinoma	Pembrolizumab	*MLH1*	H	N/A	N	At the time of the report, the patient has been treated with pembrolizumab for 1 year, without any evidence of tumor progression

MSI, microsatellite instability; MSI-H, microsatellite instability-high; TMB, tumor mutational burden; cCR, clinical complete response; PR, partial response; ICI, immune checkpoint inhibitor; N/A, not applicable.

Although the response to ICIs has not yet been fully understood. One approach to explanation is as follows: the increased rate of neoantigens can cause an immune response and an upregulation of PD-1 ligands within the immunogenic tumor environment (20).

The most recent guidelines from the National Comprehensive Cancer Network (NCCN) and the European Society for Medical Oncology (ESMO) recommend the use of pembrolizumab as a first-line treatment for patients with dMMR/MSI-H metastatic colorectal cancer (21, 22). These guidelines emphasize the importance of genetic testing for LS and other mismatch repair deficiencies to guide immunotherapy decisions. Moreover, the guidelines suggest close monitoring for treatment-related adverse events and advocate for a multidisciplinary approach to manage these patients effectively.

In our case, the patient with LS received PD-1 inhibitor monotherapy (pembrolizumab) after liver metastases. He achieved clinical complete response (cCR) until now, while the TRAE also occurred after 17 cycles of pembrolizumab. A body of studies reported that the TRAEs are predictive biomarkers for ICI efficacy, including bystander inflammation of off-target organs due to activated infiltrating T cells. ICI-associated diabetes could be caused by the autoreactive CD8+ T-cells, which are infiltrating pancreatic islets and destroy insulin-producing beta cells (23). Most of the patients present with diabetic ketoacidosis characterized by absent C-peptide, causing the decline of the insulin production, as was observed in our patient. He is still receiving pembrolizumab treatment every 3 weeks as maintenance therapy under regularly insulin treatment.

Of course, our case also raises a question about the duration of immunotherapy, especially those with processable TRAEs. In our case, pembrolizumab was administered for 2 years. The median duration of treatment with Pembrolizumab is 11.1 months, with the longest duration reaching 30.6 months; however, the majority of patients complete their treatment within two years (24). Ongoing clinical trials with checkpoint inhibitors in dMMR/MSI-H mCRC are needed to generate more data on treatment outcomes. Meanwhile, we believe that our observation is informative for clinicians taking care of patients with mCRC because of their genetic predisposition to LS, and get a potentially significant clinical benefit of checkpoint inhibitors used as single agents early in the dMMR/MSI-H hepatic metastasis of colonic carcinoma and LS.

## Data Availability

The raw data supporting the conclusions of this article will be made available by the authors, without undue reservation.

## References

[1] Sinicrope FA . Lynch syndrome-associated colorectal cancer. N Engl J Med. (2018) 379:764–73. doi: 10.1056/NEJMcp1714533 30134129

[2] Jin Z Sinicrope FA . Mismatch repair-deficient colorectal cancer: building on checkpoint blockade. J Clin Oncol. (2022) 40:2735–50. doi: 10.1200/JCO.21.02691 PMC9390830 35649217

[3] Boukouris AE Theochari M Stefanou D Papalambros A Felekouras E Gogas H . Latest evidence on immune checkpoint inhibitors in metastatic colorectal cancer: A 2022 update. Crit Rev Oncol Hematol. (2022) 173:103663. doi: 10.1016/j.critrevonc.2022.103663 35351582

[4] Taieb J Svrcek M Cohen R Basile D Tougeron D Phelip JM . Deficient mismatch repair/microsatellite unstable colorectal cancer: Diagnosis, prognosis and treatment. Eur J Cancer. (2022) 175:136–57. doi: 10.1016/j.ejca.2022.07.020 36115290

[5] Umar A Boland CR Terdiman JP Syngal S de la Chapelle A Ruschoff J . Revised Bethesda Guidelines for hereditary nonpolyposis colorectal cancer (Lynch syndrome) and microsatellite instability. J Natl Cancer Inst. (2004) 96:261–8. doi: 10.1093/jnci/djh034 PMC2933058 14970275

[6] Ascrizzi S Arillotta GM Grillone K Carida G Signorelli S Ali A . Lynch syndrome biopathology and treatment: the potential role of microRNAs in clinical practice. Cancers (Basel). (2023) 15(15):3930. doi: 10.3390/cancers15153930 37568746 PMC10417124

[7] Shulman K Barnett-Griness O Friedman V Greenson JK Gruber SB Lejbkowicz F . Outcomes of chemotherapy for microsatellite instable-high metastatic colorectal cancers. JCO Precis Oncol. (2018) 2:PO.17.00253. doi: 10.1200/PO.17.00253 32913995 PMC7446482

[8] Xu Y Li Q Zhao J Ni X Li P Hu W . Case report: Complete response to pembrolizumab in a liver metastatic colon adenocarcinoma patient with a novel likely pathogenic germline MSH2 mutation. Front Immunol. (2022) 13:1064488. doi: 10.3389/fimmu.2022.1064488 36518767 PMC9742472

[9] Keane F Yogiaveetil E Kezlarian B Lagratta M Segal NH Abou-Alfa G . Immune checkpoint blockade induced sarcoid-like reaction mimicking progression of disease in a patient with microsatellite instable colorectal cancer: case report and review of the literature. J Gastrointest Oncol. (2024) 15:500–7. doi: 10.21037/jgo PMC10932687 38482249

[10] Keating M Giscombe L Tannous T Hartshorn K . Prolonged treatment response to pembrolizumab in a patient with pretreated metastatic colon cancer and lynch syndrome. Case Rep Oncol Med. (2019) 2019:3847672. doi: 10.1155/2019/3847672 31565451 PMC6745160

[11] Krekeler C Wethmar K Mikesch JH Kerkhoff A Menck K Lenz G . Complete metabolic response to combined immune checkpoint inhibition after progression of metastatic colorectal cancer on pembrolizumab: A case report. Int J Mol Sci. (2023) 24(15):12056. doi: 10.3390/ijms241512056 37569431 PMC10418401

[12] Yang J Bi F Gou H . Complete pathologic response after concurrent treatment with pembrolizumab and radiotherapy in metastatic colorectal cancer: A case report. Onco Targets Ther. (2021) 14:2555–61. doi: 10.2147/OTT.S298333 PMC8053529 33880034

[13] Feng Y Cao Y Yuan M Chen R Ji X Hu X . Different responses to anti-programmed cell death protein 1 (PD-1) immunotherapy in a patient with Lynch syndrome and metachronous urothelial and colon cancer: A case report. Oncol Lett. (2019) 18:5085–90. doi: 10.3892/ol PMC6781514 31612019

[14] Salman P Panay S Fernandez R Mahave M Soza-Ried C . Evidence of response to pembrolizumab in a patient with Lynch syndrome-related metastatic colon cancer. Onco Targets Ther. (2018) 11:7295–300. doi: 10.2147/OTT PMC6205820 30425520

[15] Patil NR Khan GN . Exceptional response to A single cycle of immunotherapy in a lynch syndrome patient with metastatic pancreatic adenocarcinoma. Am J Case Rep. (2020) 21:e923803. doi: 10.12659/AJCR.923803 32658872 PMC7377526

[16] Nakase K Matsuda R Sasaki S Nakagawa I . Lynch syndrome-associated glioblastoma treated with concomitant chemoradiotherapy and immune checkpoint inhibitors: case report and review of literature. Brain Tumor Res Treat. (2024) 12:70–4. doi: 10.14791/btrt.2023.0042 PMC10864134 38317491

[17] Djerroudi L Masliah-Planchon J Brisse HJ El Zein S Helfre S Tzanis D . Metastatic Malignant perivascular epithelioid cell tumors with microsatellite instability within lynch syndrome successfully treated with anti-PD1 pembrolizumab. JCO Precis Oncol. (2023) 7:e2200627. doi: 10.1200/PO.22.00627 36716416 PMC9928971

[18] Andre T Shiu KK Kim TW Jensen BV Jensen LH Punt C . Pembrolizumab in microsatellite-Instability-High advanced colorectal cancer. N Engl J Med. (2020) 383:2207–18. doi: 10.1056/NEJMoa2017699 33264544

[19] Le DT Durham JN Smith KN Wang H Bartlett BR Aulakh LK . Mismatch repair deficiency predicts response of solid tumors to PD-1 blockade. Science. (2017) 357:409–13. doi: 10.1126/science.aan6733 PMC5576142 28596308

[20] Roudko V Cimen Bozkus C Greenbaum B Lucas A Samstein R Bhardwaj N . Lynch syndrome and MSI-H cancers: from mechanisms to “Off-the-shelf” Cancer vaccines. Front Immunol. (2021) 12:757804. doi: 10.3389/fimmu.2021.757804 34630437 PMC8498209

[21] Benson AB Venook AP Adam M Chang G Chen YJ Ciombor KK . Colon cancer, version 3.2024, NCCN clinical practice guidelines in oncology. J Natl Compr Canc Netw. (2024) 22(2 D):e240029. doi: 10.6004/jnccn.2024.0029 38862008

[22] Yoshino T Cervantes A Bando H Martinelli E Oki E Xu RH . Pan-Asian adapted ESMO Clinical Practice Guidelines for the diagnosis, treatment and follow-up of patients with metastatic colorectal cancer. ESMO Open. (2023) 8:101558. doi: 10.1016/j.esmoop.2023.101558 37236086 PMC10220270

[23] Tsang ES Walker EJ Carnevale J Fisher GA Ko AH . Durable response after immune checkpoint inhibitor-related diabetes in mismatch repair-deficient pancreatic cancer. Immunotherapy. (2021) 13:1249–54. doi: 10.2217/imt-2021-0008 34338034

[24] Casak SJ Marcus L Fashoyin-Aje L Mushti SL Cheng J Shen YL . FDA approval summary: pembrolizumab for the first-line treatment of patients with MSI-H/dMMR advanced unresectable or metastatic colorectal carcinoma. Clin Cancer Res. (2021) 27:4680–4. doi: 10.1158/1078-0432.CCR-21-0557 PMC8416693 33846198

